# Death of Professor Horner

**Published:** 1853-04

**Authors:** 


### THE WESTERN JOURNAL.

Third Series.—Vol. XI.—No. IV.

LOUISVILLE, APRIL 1, 1853.

### DEATH OF PROFESSOR HORNER.

The eminent Professor of Anatomy in the University of Pennsylvania died, on the 13th of March last, in the 60th year of his age. The name of Dr. Horner was known to every practitioner of medicine in America; and his amiable qualities in private life had endeared him to a great number who have been related to him as pupils. We have heard many who had been his private students speak of him with a warmth of affection, that showed how kind and considerate was his deportment as a preceptor and friend. We learn from a biographical sketch of Dr. Horner, in the Medical Examiner, that he was born at Warrenton, Fauquer county, Va., on the 31st of June, 1793. At the age of 17, he commenced the study of medicine with Dr. John Spence, a Scotch physician of considerable reputation.

In the autumn of 1812, he attended his first course of lectures in the University of Pennsylvania, and soon began to evince a taste for that branch of the profession in which he subsequently became so distinguished, being occasionally employed in assisting the Demonstrator of Anatomy in the preparation of Prof. Wistar's lecture. He obtained a commission, the summer following, as Surgeon's Mate in the Army of the United States, and served in a regiment stationed at Fort Mifflin, near Philadelphia. He took his degree at the University in the spring of 1814, and was ordered, in the summer of that year, to the Niagara frontier, where he was actively employed until the close of the war After the peace in 1815, he was stationed for a short time at Norfolk, but, resigning his commission soon, he returned to his native town, and commenced the practice of his profession. In the fall of the same year he removed to Philadelphia, where he remained to the end of his life, thirty-three years having been spent in the University of Pennsylvania, as Professor of Anatomy, Demonstrator, and adjunct of his great predecessor, Dr. Physick. His rise, in Philadelphia, was very rapid, and was wholly independent of extraneous circumstances. He was a stranger, without interest or influence, and yet in two years he was made Demonstrator of Anatomy, and three years afterwards adjunct professor of the same branch. On the resignation of Dr. Physick, in 1831, he was chosen professor of Anatomy, in which capacity he continued to act until wtthin a short period of his death.

Professor Horner was a laborious man. He collected, during his long connexion with the University an anatomical cabinet, said to be one of the most splendid and complete in the world. He was an accurate and faithful, rather than brilliant or impulsive teacher. He was clear, simple, and perspicuous, without earnestness or eloquence. He relied on the importance of his subject, and the thoroughness with which he presented it, rather than on grace of manner or beauty of diction; and his happy demonstrations were generally successful in arresting and fixing the attention of his pupils. He commanded the respect and confidence of his class. In every personal and professional relation he was without reproach, and leaves an unstained reputation behind him. He had wisely attended to the substantial rewards of his profession while enjoying its honors, and by his own unaided exertions had secured an ample fortune. "No one," says the writer of the sketch in the Medical Examiner, "could have closed his life under more consolations; and that greatest and best of consolations, a firm Christian hope, had been long and well secured." The same writer adds the following interesting particulars relative to the disease which terminated the life of this eminent and good man:

"Though up to a short period of his death, Dr. Horner continued in the steady discharge of his professional and other duties, he had for years suffered from dyspnœa, palpitation, and other symptoms, which left litle doubt that he labored under cardiac disorder; and the emaciation of his frame made it evident that it was producing serious derangement of the functions of nutrition. For some weeks preceding his death, dropsical effusion had appeared. And, though within a day or two of his death, he was able to participate in the examination of students for degrees, yet we believe that neither himself nor his family entertained any hope that his life could be long protracted. The immediate termination, was, however, somewhat unexpected.

"The *post-mortem* examination confirmed the diagnosis of disease of the heart. It was found very considerably hypertrophied and enlarged, being five and a half inches from the apex to the origin of the pulmonary artery, five and three-quarter inches in diameter, and thirteen and a half inches in circumference at its base. The tricuspid and mitral valves were healthy. The arch of the aorta was dilated and thickly ossified. There was also recent peritonitis, with streaks of fresh coagulable lymph over the peritoneal surface of the intestines. Perforation of the stomach or bowels had been suspected, as the cause of the peritonitis, but no traces of this lesion were found."

